# The new gamma interferon (IFN-γ) algorithm for tuberculosis diagnosis in cynomolgus macaques

**DOI:** 10.1371/journal.pone.0302349

**Published:** 2024-12-16

**Authors:** Saradee Warit, Suthirote Meesawat, Pattsarun Cheawchanlertfa, Nampueng Makhao, Prapaporn Srilohasin, Machamon Kaewparuehaschai, Kirana Noradechanon, Areeya Pomcoke, Taratorn Kemthong, Therdsak Prammananan, Reka Kanitpun, Tanapat Palaga, Suchinda Malaivijitnond, Angkana Chaiprasert

**Affiliations:** 1 Industrial Tuberculosis Team, Industrial Medical Molecular Biotechnology Research Group, BIOTEC, National Science and Technology Development Agency, Pathum Thani, Thailand; 2 National Primate Research Center of Thailand- Chulalongkorn University, Saraburi, Thailand; 3 Office of Research, Faculty of Medicine Siriraj Hospital, Mahidol University, Bangkok, Thailand; 4 Department of Microbiology, Faculty of Medicine Siriraj Hospital, Mahidol University, Bangkok, Thailand; 5 Department of National Parks, Wildlife and Plant Conservation (DNP), Bangkok, Thailand; 6 National Institute of Animal Health (NIAH), Kaset Klang, Bangkok, Thailand; 7 Department of Microbiology, Faculty of Science, Chulalongkorn University, Bangkok, Thailand; 8 Department of Biology, Faculty of Science, Chulalongkorn University, Bangkok, Thailand; Stellenbosch University, SOUTH AFRICA

## Abstract

Tuberculosis (TB) is the first infectious disease to be screened-out from specified pathogen-free cynomolgus macaques (*Macaca fascicularis*; Mf) using in human pharmaceutical testing. Being in either latent or active stage after exposure to the *Mycobacterium tuberculosis* complex (MTBC), the monkey gamma-interferon release assay (mIGRA) was previously introduced for early TB detection. However, a notable incidence of indeterminate results was observed. In this study, we compared two positive mitogen references, phytohemagglutinin (PHA) that is used in the QuantiFERON-TB Gold Plus kit (QFT-PHA) and a combination of Concanavalin A and Pokeweed mitogen (ConA+PWM), in a cohort of 316 MTBC-exposed Mf. Following a 29-month follow-up of 100 selected animals, we established a new mIGRA interpretation algorithm that demonstrated a significant shift in the negative and indeterminate cases regardless of whether the QFT-PHA or ConA+PWM was used as a mitogen. That is, if the OD_NIL_ value was ≤0.18, OD_MIT-NIL_ > OD_NIL_, and the OD_TB1/2-NIL_ were ≥0.05 and ≥25% of individual OD_NIL_, the mIGRA result was interpreted as ‘positive’. If the OD_NIL_ value was ≤0.18, OD_MIT-NIL_ > OD_NIL_, and the OD_TB-NIL_ was <0.05, the mIGRA result was interpreted as ‘negative’. If the OD_NIL_ value was >0.18 or the OD of mitogen references [OD_(QFT-PHA)_ and OD_(ConA+PWM)_] were ≤0.18, the mIGRA result was interpreted as ‘indeterminate’. As a result, negative cases increased by 10–50%, indeterminate cases decreased by 40–80%, and the number of TB-positive cases remained unchanged. Our findings highlight the critical role of mitogens as positive controls in mIGRA interpretation. This study provides the mIGRA value for the TB screening of cynomolgus macaques that enables the identification of true positive and suspicious TB cases for quarantine programs.

## Introduction

With their close phylogenetic relationships and physiological similarities to humans, nonhuman primates (NHPs) have been commonly used to test the safety and efficacy of human pharmaceuticals [1–4]. Specified pathogen-free NHPs are highly demanded for these tests. Tuberculosis (TB)-free is an important requirement for imported laboratory NHPs, including cynomolgus macaque (*Macaca fascicularis*), for the Association of Primate Veterinarians (APV) and the Centers for Disease Control and Prevention in the United States and Canada [3, 5].

TB is a chronic bacterial infection caused by a group of *Mycobacterium tuberculosis* complex (MTBC), of which the species of greatest concern are *M*. *tuberculosis* (*M*.*tb*) and *M*. *bovis* [6, 7]. Aerosol transmission is the main factor in the outbreak of the disease. Like humans, after TB infection, cynomolgus macaque could be latent, subclinical, or active depending on the immune system of the host [8]. Therefore, early detection of TB is a primary requirement. A TB testing algorithm that incorporates multiple assays, for example, culture, molecular technique, antibody blood test, tuberculin skin test (TST), and interferon-gamma release assay (IGRA), was suggested to improve overall sensitivity and specificity [9–11]. MTBC culture (directly detected MTBC) is supposed to be a gold standard method in humans, but it was inconvenient for routine screening in a large number of captive monkeys. Molecular techniques such as IS*6110* nested PCR or GeneXpert took a shorter time, but could not detect the immune response. Although the antibody blood test such as antibody-ELISA was developed, the humoral-mediated immune response appeared after the cell-mediated immune response, leading the TST and/or IGRA to be kept as preferable methods in many primate facilities [11, 12].

The *in vivo* TST test is a classical tool for TB detection in NHPs. The test uses purified protein derivatives (PPD) to stimulate host immune cells to secrete cytokines by intradermal injection into the eyelid. Swelling and redness 24, 48, and 72 hours after injection are monitored, meaning that at least two visits are needed. Other limitations of the TST are, for example, an expert demand in steps of observation and interpretation and a shortage of PPD. Thus, the IGRA is introduced. The IGRA blood test is convenient and does not require repeated handling of animals or extended intervals prior to retesting [13]. Two commercial IGRA kits, Quantiferon-plus (QFT-plus) and ELISpot kits, used two specific mycobacterial peptides of early secreted antigenic target 6 kDa (ESAT6) and 10 kDa culture filtrate antigen (CFP-10) to stimulate blood immune cells (mainly lymphocytes) and measured IFN-γ levels. Both ESAT6 and CFP10 are the most popular biomarkers due to their role in mycobacterial virulence and absence in the *M*. *bovis* BCG-vaccine strain [13–15]. Interpretation was carried out by comparing with negative (no mitogen added) and positive (mitogen added) controls. Recently, our team has introduced monkey IGRA (mIGRA) for the detection of TB in naturally TB-infected macaques by combining human blood IGRA tubes (the QFT plus kit) and the monkey IFN-γ ELISA kit together [16]. However, many indeterminate results were unexpectedly encountered. Two factors were considered, high IFN-γ background level (NIL) and low mitogen (MIT) response. Aberrant IFN-γ expression of NIL without any stimulation is probably associated with autoinflammatory, autoimmune diseases, and viral infection of the hosts, which could not be directly correlated with TB infection [17]. The low response to mitogen stimulation should be a mitogen of choice, such as a phytohemagglutinin (PHA) used in the QFT-plus kit.

Mitogens are nonspecific stimulants of immune cells that can activate various lymphocyte subpopulations to secrete cytokines such as IFN-γ [18, 19]. In the IGRA test, a mitogen serves as a positive control for the nonspecific T-cell response and reflects the response of active T lymphocytes in a host sample [20]. Commercially, commonly used mitogens are phytohemagglutinin (PHA), concanavalin A (Con A), and pokeweed (PWM). PHA, the lectin extract from the red kidney bean (*Phaseolus vulgaris*), contains potent, cell agglutinating and mitogenic activities [21]. PHA binds to the membranes of T cells and stimulates metabolic activity, cell agglutination, and mitogenic activities [22, 23]. Con A is another plant lectin (carbohydrate-binding protein) originally extracted from the jack bean (*Canavalia ensiformis*) and is known for its ability to stimulate mouse T-cell subsets, giving rise to four functionally distinct T cell populations, including precursors to regulatory T cells [24]. In addition, Con A does not have any mitogenic effect on B cells [25]. PWM, a lectin mitogen derived from the roots of *Phytolacca americana*, functions as a mitotic stimulus for the division of lymphocytes and specifically induces the proliferation of B cells, plasma cells, and T cells in mice and humans [26–28]. Since no reports of the mitogenic effect and secreted IFN-γ levels of the IGRA test were found in cynomolgus macaques, this study was performed to compare 8 sets of mitogens on IFN-γ stimulation and to establish a new mIGRA algorithm for the interpretation of TB infection.

## Materials and methods

### Animal ethics and permit

This study was carried out in strictly accordance with the recommendations in the Guide for the Care and Use of Laboratory Animals Committees of Mahidol University and the Department of Nationals, Wildlife and Plant Conservation of Thailand. The experimental procedures and protocols working with captive macaques at the Krabok-Koo Wildlife Rescue Center (KBK) were approved by the Committee on the Ethics of Animal Experiments of the Mahidol University (Protocol review number: 009/2564).

### Animal husbandry, welfare, and blood collection

The subjects of this study were 316 cynomolgus macaques (*Macaca fascicularis*) socially housed in gang cages (4 x 10 x 4 m for W x L x H) at Krabok-Koo Wildlife Rescue Center (KBK), Ta Takiap District, Chachoengsao Province, eastern Thailand, which is under the authority of the Department of National Parks, Wildlife and Plant Conservation of Thailand (DNP). The housing system was an outdoor colony with uncontrolled natural environments. Some animals were housed in the same cage previously reported cynomolgus macaques naturally infected with *Mycobacterium tuberculosis* complex (MTBC) [16], while others are housed in the vicinity. They were 250 (79%) males and 66 (21%) females ranging from 2 to 15 years of age with a body weight of 2–10 kg. The macaques were fed with cooked rice in the morning (09.00–10.00 h), fresh fruits and vegetables, i.e., melon, banana, guava, dragon fruit, and cucumber, in the afternoon (14.00–15.00 h) and drinking water *ad libitum*. Behaviors and appetite were observed twice daily during food provisioning without monkey anesthetization. The excrement was cleaned twice a week. In each round of blood specimen collection, when the monkeys were anesthetized, the TB results from the previous round of the study were used to decide which monkeys to be returned to their home cage (TB-negative monkeys) and which monkeys to be transferred to an isolated individual cage (1 x 3 x 1.8 m for W x L x H) located approximately 400-meter from the gang cages. The animal care protocol was similar in both the gang cages and individual cages.

The monkeys were anesthetized with a mixture of zoletil (3–5 mg per kg) and dexmedetomidine hydrochloride (0.03–0.05 mg per kg), collected blood samples, and examined physical conditions. Blood was collected by femoral venipuncture, transferred to lithium-heparin tubes (cat. no. care-LIT4, Carestainer, China), and IFN-γ levels were determined using the two-step IGRA test (see details below).

Blood samples were first collected from 12 monkeys (7 males and 5 females), aged 5.5–13 years and 3–11 kg of body weight, for screening of 5 mitogen stimulations on IFN-γ levels. They were selected from 316 monkeys according to the criteria that they were negative for all TB tests, including TST, IGRA, ELISA (for ESAT6 and CFP10 antibodies), and GeneXpert ultra. One of the 5 mitogens that showed the highest IFN-γ level simulation was selected and tested again in all 316 monkeys.

Among 316 monkeys, 100 monkeys suspected of TB (42 individuals were tested positive for either TST, IGRA, or GeneXpert ultra, 14 individuals were in the same cage with a dead animal infected with MTBC, and the remaining 44 individuals were tested negative for TST, IGRA and GeneXpert ultra) were selected and followed for 8 rounds at 0, 4, 7, 14, 17, 20, 26 and 29 months (M0, M4, M7, M14, M17, M20, M26, and M29, respectively). These animals were used in the prospective study to determine mIGRA cutoff values and to establish a new mIGRA interpretation algorithm. The results of M11 and M23 were excluded in this experiment because of the limitation of the accession of plasma specimens.

### Mitogens

The mitogens used were phytohemagglutinin (PHA) from *Phaseolus vulgaris* (cat no. L1668, Sigma), concanavalin A (Con A) from *Canavalia ensiformis* (cat no. C5275, Sigma), lectin or pokeweed mitogen (PWM) from *Phytolacca americana* (cat no. L8777, Sigma). Commercially available phytohemagglutinin from the QuantiFERON-TB Gold- Plus kit (QFT-Plus; Catalog no. 622536, QIAGEN, USA) with an unknown concentration in the mitogen tube (QFT-PHA) was also used as a reference.

### Comparison of mitogen stimulations on IFN-γ secretion

Among the 12 monkeys with negative TB selected, stimulations of 4 mitogen sets [PHA (20 μg/ml), Con A (20 μg/ml), PWM (20 μg/ml), Con A+PWM (20 μg/ml each in a ratio of 1: 1)] were compared to that of the QFT-PHA mitogen of the QFT-plus kit. Each 0.5 ml of blood was transferred to 4 mitogen tubes (PHA, Con A, PWM, and Con A+PWM) and 1.0 ml of blood was transferred to the QFT-PHA tube following the instruction of the QFT-plus kit within 16 hours after blood sampling. After stimulation, IFN-γ levels were determined by IFN-γ ELISA (see the details below), and optical density (OD) values at a wavelength of 450 nm were read. The OD_450_ value of the plasma background (OD_NIL_) was used to subtract the OD_450_ value of the mitogen (OD_MIT_), and the OD_MIT-NIL_ of the 5 mitogen sets was compared. Since the OD_MIT-NIL_ value of ConA+PWM was the highest (see Results), only the mitogen stimulation tests between ConA+PWM and QFT-PHA were repeated in all 316 monkeys.

### Plasma IFN-γ determination using the mIGRA test

In this study, the two-step mIGRA test; blood stimulation and measurement of IFN-γ level was used. Five ml of blood was collected from each animal.

#### Blood stimulation

One ml of heparinized whole blood was aliquoted in each of the 5-format tubes (QFT-NIL, QFT-TB1, QFT-TB2, QFT-PHA, and Con A+PWM) within 16 hours after blood sampling. All tubes were vertically tilted to mix antigens in the blood prior to incubation at 37°C for 20 hours. After incubation, plasma samples were harvested and stored at −80 °C before the monkey IFN-γ ELISA assay (see below). All steps were carried out under the class II biosafety cabinet (BSC) (Model NU-440-600E, Nuaire, USA).

#### Determination of plasma IFN-γ levels

Plasma sample was diluted with ELISA diluent (catalog number 3652-D2, Mabtech, Sweden) in a ratio of 1:2. The diluted plasma was determined IFN-γ levels using a commercial monkey IFN-γ ELISApro kit (catalog no. M4210M-1HP-10, Mabtech AB, Sweden), containing mAb MT126L, mAb7-B6-1-biotin and human IFN-γ standards. Monkey IFN-γ levels were determined by measuring the OD at a wavelength of 450 nm (OD_450_). OD_450_ values of TB1, TB2, QFT-PHA, and Con A+PWM were subtracted with the OD_450_ value of the IFN-γ background (OD_NIL_) as OD_TB1-NIL_, OD_TB2-NIL_, and OD_MIT-NIL_ before read-out. Before the interpretation of the mIGRA result, the cutoff values and the criteria were established, as described in the result section.

### Statistical analysis

The normal distribution of plasma IFN-γ levels was tested. If the non-normal distribution was detected, the IFN-γ levels were analyzed using Friedman’s tests. All statistical analysis and graphical presentations were performed using GraphPad Prism 9 software version 9.0.0 (GraphPad, SanDiego, CA, USA). *p*-values less than 0.05 were considered significant differences.

## Results

### Comparison of mitogen stimulation on IFN-γ secretion of monkey blood cells

**Fig 1:**
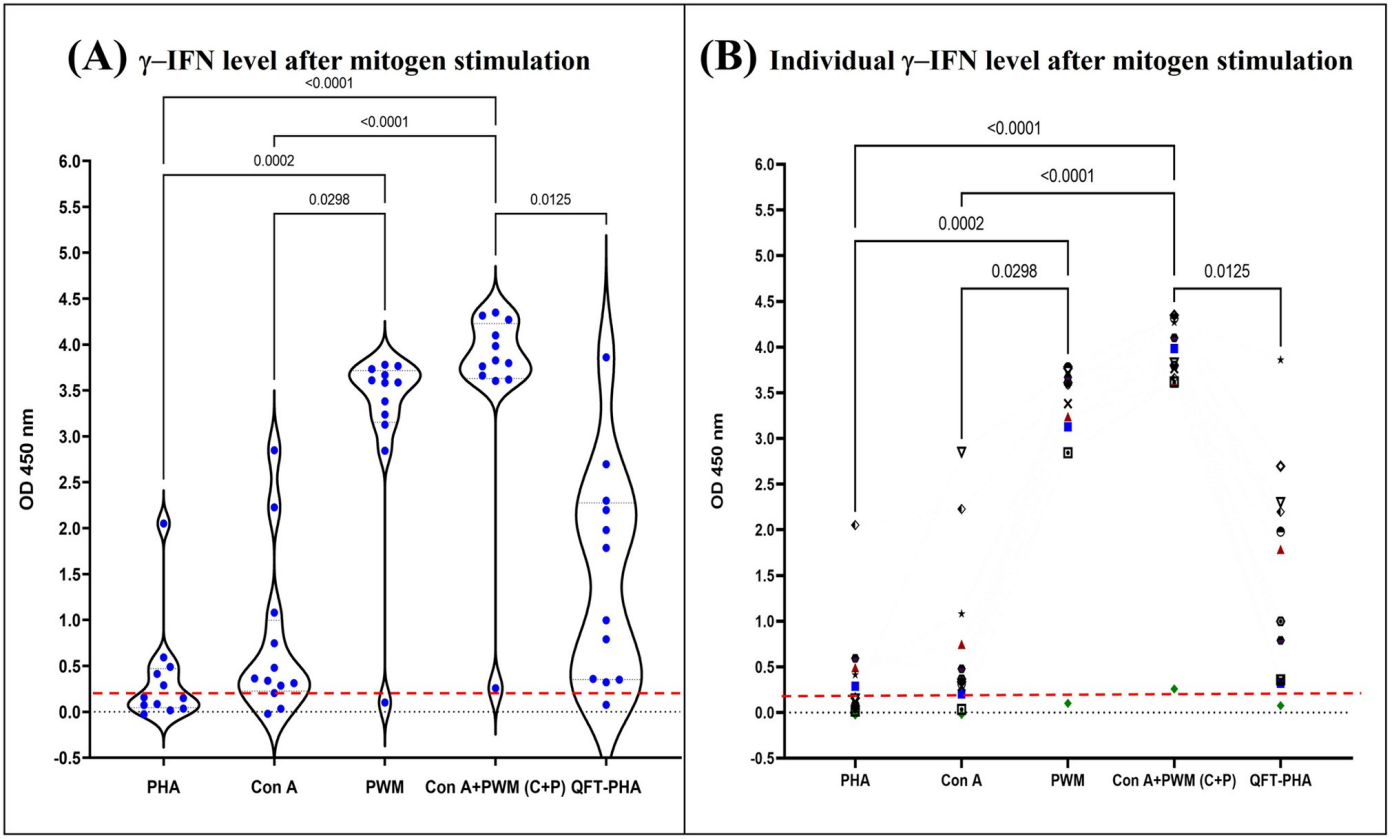
IFN-γ values as OD_MIT-NIL_, of 12 selected cynomolgus macaques after stimulation with prepared PHA (20 μg/ml), Con A (20 μg/ml), PWM (20 μg/ml), a mixture of Con A and PWM (C+P; 20 μg/ml each), and (QFT-PHA) and subtraction of the blank and plasma background. **Left panel:** The OD_450_ values of IFN-γ after mitogen stimulation for 20h at 37°C. All OD_450_ values are also marked as spots. **Right panel:** Individual OD_450_ values of IFN-γ levels responding to 5 mitogen sets were obtained from 12 cynomolgus macaques. The red line indicates the 95^th^ percentile of OD_NIL_ (= 0.18).

When comparing the OD_MIT-NIL_ of all 5 mitogen sets (PHA, Con A, PWM, Con A+PWM, and QFT-PHA) of 12 monkeys, it was found that the OD_MIT-NIL_ value of Con A+PWM was significantly higher than the other 4 mitogen stimulations as follows; Con A+PWM > PWM > QFT-PHA > Con A > PHA (Fig 1 and S1 Table). Monkey No. KBK114 showed low responses in all 5 mitogen stimulations (OD_450_ values ranging (-0.027)–0.258), while Monkey No. KBK292 showed high responses in all 5 mitogen stimulations (OD_450_ values ranging 0.414–4.270). However, the OD_MIT-NIL_ values by Con A+PWM stimulation remained higher than other mitogen stimulations in these two monkeys. Note that 12 macaques gave higher OD_450_ values for QFT-PHA stimulation than for PHA (S1 Table).

**Fig 2:**
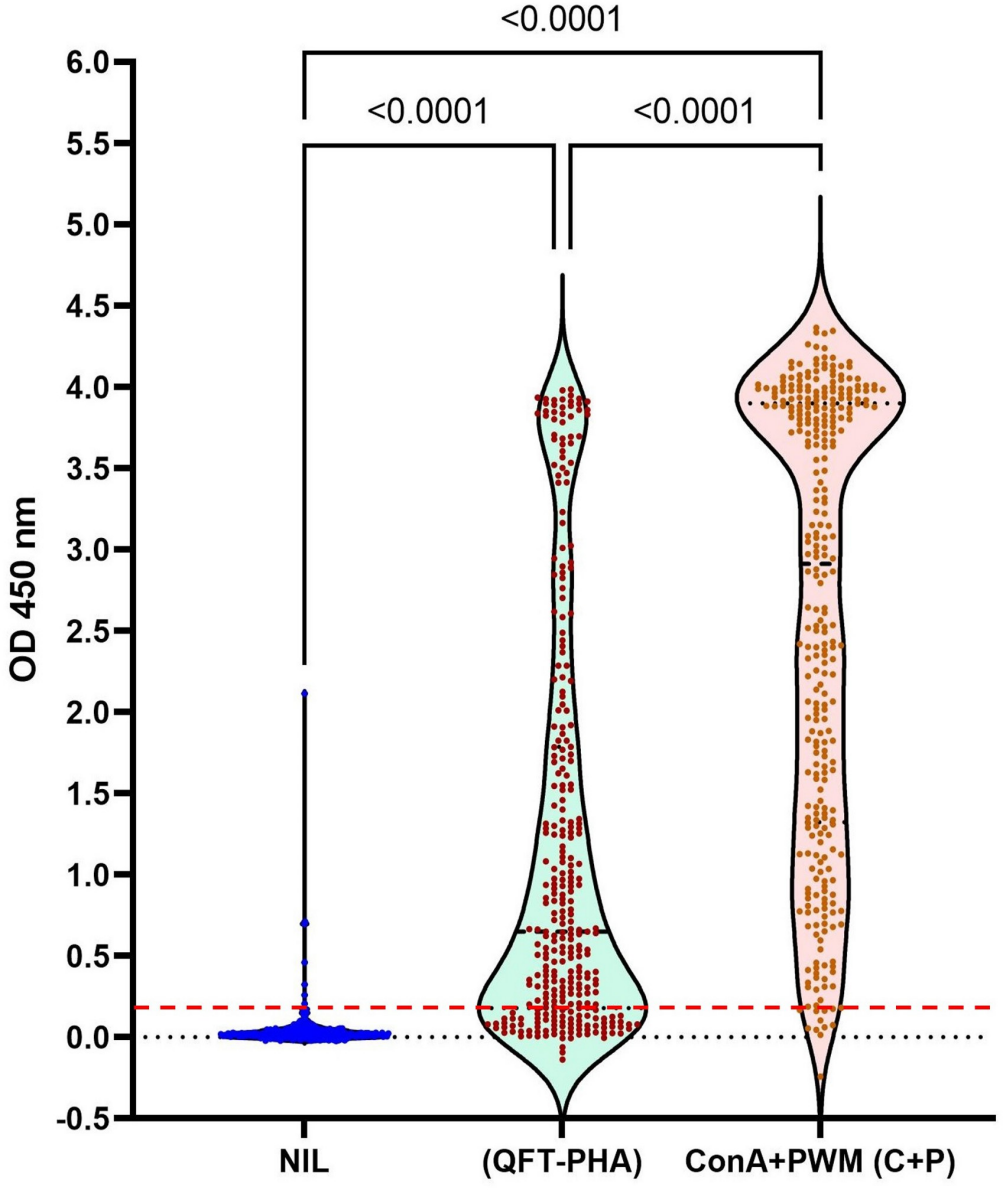
All IFN-γ levels as OD_450_ values obtained from whole blood immune cells from 316 naturally TB-contact cynomolgus macaques after stimulation with QFT-PHA and Con A+PWM (C+P) mitogens and without any stimulator (The NIL tube). All minimum and maximum data values were plotted together with the mean (+) and median (a line) values in a box. The red line indicated the 95^th^ percentile of OD_NIL_ (= 0.18).

To confirm that mitogen selection used for TB diagnosis with mIGRA is one of the key factors to be considered for mIGRA interpretation, mitogen stimulation tests comparing between QFT-PHA and Con A+PWM were done in a cohort—316 cynomolgus macaques. Using QFT-PHA as a mitogen, monkeys showed OD_MIT-NIL_ values that ranged (-0.14)–3.99 (mean ± SD = 1.18±1.26). If the mixture of Con A+PWM was used as the mitogen, the OD_MIL-NIL_ values ranged by (-0.24)–4.36 (mean ± SD = 2.59±1.37) (Fig 2). Comparison of the IFN-γ response between QFT-PHA and Con A+PWM stimulations, 93.7% of the animals (296/316) showed the higher value of IFN-γ to the Con A+PWM mitogen than that of QFT-PHA (*p*-value < 0.0001), while 6% of the animals (19/316) showed the opposite direction. Surprisingly, 0.3% of the animals (1/316) did not show any response to any mitogen controls. Furthermore, it was found that 72 (22.8%) and 12 (3.8%) animals gave the OD_MIT-NIL_ values less or equal to 0.18 when their immune cells were stimulated with QFT-PHA and Con A+PWM, respectively. Moreover, there were 9 (2.8%) animals, giving the OD_NIL_ values higher than 0.18. Nevertheless, this suggests that a combination of Con A+PWM should be used as a mitogen positive control to determine host immune status through plasma IFN-γ level after blood stimulation in cynomolgus monkeys. Thus, the mixture of Con A+PWM mitogens was selected for the next assays and compared to QFT-PHA when reporting the mIGRA interpretation results.

### Determination of mIGRA cutoff values and a new algorithm to report TB infection in cynomolgus macaques

The IFN-γ OD_450_ values were obtained from 5-format tubes (NIL, TB1, TB2, QFT-PHA and Con A+PWM), calculated and presented as OD_NIL_, OD_TB1-NIL_, OD_TB2-NIL_, OD_(QFT-PHA)-NIL_ and OD_(ConA+PWM)-NIL_ values. Using standards in a concentration of 7.87, 15.75, 31,25 and 62.5 pg/ml according to the monkey γ-IFN ELISA kit manual (Cat. No. 3421M-1H-20, Mabtech, Sweden), the percentage of the inter-assay and intra-assay coefficient of variation (%CV) were determined and found to be in a range of 23–26% and 0–15%, respectively. To determine the cutoff value, the OD_450_ values obtained from 100 selected cynomolgus macaques, which were followed approximately every 3 months for 8 rounds (M0, M4, M7, M14, M17, M20, M26, and M29), total 717 values, were pooled and analyzed. Using a box plot analysis, the 95^th^ percentile of OD_NIL_ was calculated and found to be 0.18. Next, the OD_450_ values of TB1 and TB2 that were higher than the OD_NIL_ value (or OD_TB1-NIL_ and OD_TB2-NIL_ > OD_NIL_) were selected. The 25% (Q1) and 75% (Q3) percentile values of OD_TB1-NIL_ and OD_TB2-NIL_ and the interquartile range (IQR) values were calculated. Outlier values were identified from an upper limit value [Q3+ (1.5*IQR)] and a lower limit value [Q1-(1.5*IQR)]. As a result, the upper and lower limit values of both OD_TB1-NIL_ and OD_TB2-NIL_ were 0.05 and -0.04, respectively.

**Fig 3:**
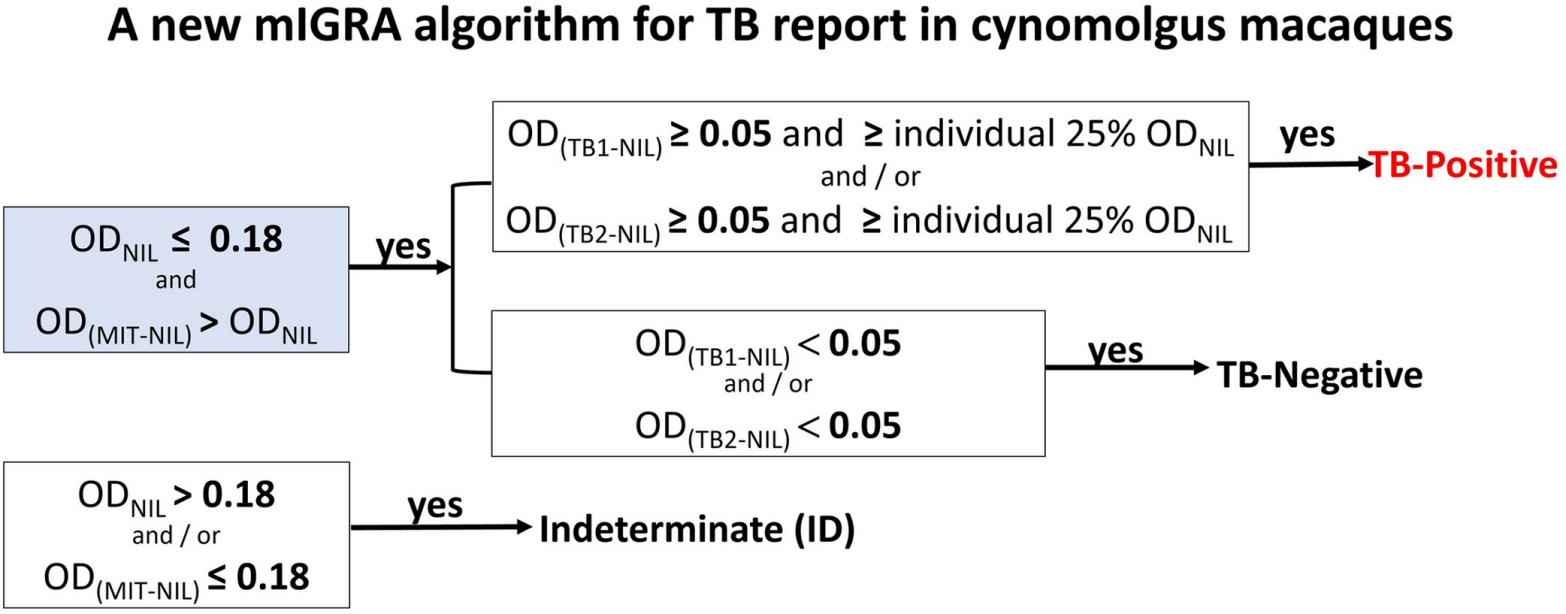
A flow chart of the new mIGRA interpretation algorithm for TB report in cynomolgus macaques using QFT plus tubes (NIL, TB1 and TB2) and a mixture of Con A and Pokeweed mitogen (Con A+PWM) as a positive mitogen control. OD is an optical density reading at wavelength of 450 nm. The TB1 tube contains ESAT6 and CFP10 peptides specific to CD4+-T cells, whereas the TB2 tube contains ESAT6 and CFP10 peptides specific to CD4+ and CD8+ -T cells. The NIL tube means that there is no mitogen stimulation. The MIT tube contains either a mitogen ConA+PWM or QFT-PHA, which was applied in this study.

Following the QFT-plus kit handout, it suggested that after stimulation, the level of IFN-γ of the TB tubes (TB1 or TB2) should be higher than 25% of the individual IFN-γ value of the NIL tube (OD_NIL_); thus, we included this value as an additional cutoff value in our study. Therefore, OD_TB1-NIL_ and OD_TB2-NIL_ were determined from the two cutoff values, 0.05 and 25% of the individual OD_NIL_ value (Fig 3). With respect to these criteria, the interpretation of the IFN-γ ELISA is as follows. If the OD_NIL_ value was ≤0.18, OD_MIT-NIL_ > OD_NIL_, and the OD_TB1/2-NIL_ was ≥0.05 and ≥25% of individual OD_NIL_, the mIGRA result was interpreted as “positive”. If the OD_NIL_ value was ≤0.18, OD_MIT-NIL_ > OD_NIL_, and the OD_TB-NIL_ was <0.05, the mIGRA result was interpreted as “negative”. If the OD_NIL_ value was >0.18, indicating the high IFN-γ background. Thus, IFN-γ secretion after stimulation with TB1 or TB2 was invalid, and the mIGRA result was interpreted as ’indeterminate’. Furthermore, if the OD of mitogen-positive controls [OD_(QFT-PHA)_ and OD_(ConA+PWM)_] were lower or equal to 0.18, the mIGRA result was also interpreted as ’indeterminate’. This indicated that host immune cells were inactive or impaired host cell-mediated immunity.

Together, a new mIGRA interpretation algorithm was proposed using the OD_450_ values (Fig 3). After considering OD_NIL_ ≤ 0.18 and OD_MIT-NIL_ > OD_NIL_, if the OD_TB1/2-NIL_ was ≥0.05 and ≥25% of the individual OD_NIL_ value, the positive was reported and likely implied the MTBC infection. If OD_TB1/2-NIL_ was <0.05, the negative was reported and likely implying non-MTBC infection. However, indeterminate (ID) was reported, if either OD_MIT-NIL_ ≤ 0.18 or OD_NIL_>0.18 was detected.

### Comparison of TB infection result between the use of QFT-PHA and Con A+PWM mitogens

After the new mIGRA interpretation algorithm was established (Fig 3), the mIGRA interpretations of the TB infection in 100 selected cynomolgus monkeys from 8-round prospective studies were done. The use of QFT-PHA and Con A+PWM as the positive mitogens was compared. As shown in Table 1, the positive cases of mIGRA were the same regardless of comparison with any mitogen type, that were 17, 16, 13, 14, 15, 13, 8, and 7 positive cases for M0, M4, M7, M14, M17, M20, M26, and M29, respectively. The interpretation of the MTBC infected stages as active-TB, latent-TB and healthy, in 100 monkeys during those 8-round studies based on our mIGRA results combining with the results of GeneXpert MTB/RIF Ultra assay [29], TST (detailed data was not shown), antibody-ELISA test (detailed data was not shown) were summarized and showed in S2 Table. Following World Health Organization (WHO) has recommended GeneXpert MTB/RIF Ultra assay for the detection of TB in humans and it showed 98.1% of specificity and 87.8% of sensitivity when tested with the retrospective TB-adult patient samples [30, 31], the results of GeneXpert MTB/RIF Ultra assay were used as a standard method for TB diagnosis and for indication of active-TB (shedding) stage in this study. Noted, the active-TB stage indicated the animals were positive with the GeneXpert MTB/RIF Ultra assay and/or positive with either one of mIGRA, TST or antibody test. The latent-TB stage indicated the animals were negative for GeneXpert MTB/RIF Ultra assay, but they were positive with either one of mIGRA, TST or antibody test, while healthy means animals were negative for all tests. To prevent the MTBC outbreak, after M4, monkeys showing the active-TB stage were separated from their gang cages and moved to a clean remote area for further investigation and management, while monkeys staying in the latent-TB stage were considered separated based on other parameters of their health status such as more than 20% weight loss.

**Table 1:** TB interpretation with the new algorithm of mIGRA using either QFT-PHA or Con A + PWM as positive mitogens. Data was obtained from the 8-round prospective studies of 100 naturally TB-contacted cynomolgus macaques in month-0 (M0), month-4 (M4), month-7 (M7), month-14 (M14), month-17 (M17), month-20 (M20), month-26 (M26), and month-29 (M29). At M4, M7, M14, M17, M20, M26, and M29, blood collection was not applicable in some monkeys because of the animal loss or animal death occurred; thus, the total studied cases were less than 100 animals. The asterisk (*) indicates 2 positive cases infected with *M*.*tb*, which died in M9-M10.

mIGRA interpretation		QFT-PHA mitogen (Cases)								Con A+PWM mitogen (Cases)							
		M0	M4	M7	M14	M17	M20	M26	M29	M0	M4	M7	M14	M17	M20	M26	M29
**Positive**		17	16*	13*	14	15	13	8	7	17	16*	13*	14	15	13	8	7
**Negative**		51	66	68	51	49	59	46	57	76	72	79	66	65	65	69	73
**Indeterminate**	OD_NIL_ value more than 0.18 (95percentile of OD_NIL_ value)	1	9	3	7	6	4	7	4	1	9	3	7	6	4	7	4
	OD_(MIT-NIL)_ value less than or equal to 0.18	31	7	12	15	16	6	23	16	6	1	1	0	0	0	0	0
***Total applicable data (cases)***		***100***	***98***	***96***	***87***	***86***	***82***	***84***	***84***	***100***	***98***	***96***	***87***	***86***	***82***	***84***	***84***
**death**		0	0	4	12	13	14	15	15	0	0	4	12	13	14	15	15
**loss**		0	0	0	1	1	2	1	1	0	0	0	1	1	2	1	1
**Not applicable**		0	2	0	0	0	2	0	0	0	2	0	0	0	2	0	0
***Total (Cases)***		*100*	*100*	*100*	*100*	*100*	*100*	*100*	*100*	*100*	*100*	*100*	*100*	*100*	*100*	*100*	*100*

The two positive mIGRA cases (KBK099, and KBK103) were later found death during M9-M10. Remarkably, in M4 and M7, they gave positive results to both GeneXpert MTB/RIF Ultra assay (S2 Table) and antibody-ELISA tests (data not shown) as well. Their lesions in the lungs and liver organs were inspected and the infection with *Mycobacterium tuberculosis* was confirmed by the culture method.

A comparative analysis of mIGRA outcomes revealed significant differences in the incidence of indeterminate cases when using of either QFT-PHA or ConA+PWM as mitogen positive controls. The lower indeterminate cases were detected when the ConA+PWM mitogens were used, compared to that of the QFT-PHA mitogen (Table 1). The ratio of the indeterminate cases of QFT-PHA: ConA+PWM were 32:7, 16:10, 15:4, 22:7, 22:6, 10:4, 30:7, and 20:4 for M0, M4, M7, M14, M17, M20, M26 and M29, respectively. Moreover, if classified the indeterminate cases into 2 categories as mentioned in the new mIGRA algorithm, it could be presented that (1) indeterminate causing from OD_NIL_>0.18 of QFT-PHA: ConA+PWM were in the same ratio as 1:1, 9:9, 3:3, 7:7, 6:6, 4:4, 7:7, and 4:4 for M0, M4, M7, M14, M17, M20, M26, and M29, respectively. While (2) indeterminate causing from OD_MIT-NIL_ ≤ OD_NIL_ of QFT-PHA: ConA+PWM were 31:6, 7:1, 12:1, 15:0, 16:0, 6:0, 23:0, and 16:0 for M0, M4, M7, M14, M17, M20, M26, and M29, respectively. Aligned with the indeterminate cases, the ratio of the negative cases of QFT-PHA: Con A+PWM were 51:76, 66:72, 68:79, 51:66, 49:65, 59:65, 46:69, and 57:73 for M0, M4, M7, M14, M17, M20, M26 and M29, respectively. Collectively, these findings indicate that the use of ConA+PWM as the positive mitogen control, along with the new mIGRA interpretation algorithm, resulted in a 40–80% reduction in indeterminate (ID) cases and a 10–50% increase in negative cases, compared to the QFT-PHA control.

To support how the mitogen-positive controls are critical for mIGRA interpretation, we applied the new algorithm (Fig 3) to assess the stages of TB infection in 316 CMs. The ratios of positive, negative, and indeterminate cases of QFT-PHA: ConA+PWM were changed to 31:31, 206:264, and 79:21, respectively. These results indicate a 28.2% increase in the ratio of negative cases and a 73.4% decrease in the ratio of indeterminate cases, while there is no difference in the ratio of positive cases between the two mitogen controls.

## Discussion

With the high demand for nonclinical drug and vaccine tests, especially during the COVID-19 pandemic, the specific pathogen-free (SPF) cynomolgus macaques were required. TB is designated as the first infectious disease that must be screened and infected macaques must be removed from the colony immediately. Among various TB detection tools, IGRA is accepted as a method to detect early TB infection and the latent period [11, 12]. As such, the monkey IGRA (mIGRA) assay was developed [16]. A major hindrance of the mIGRA assay in monkeys was the high rate of indeterminate results due to insufficient stimulation of the mitogen used in the assay. The IGRA mitogen serves as a critical reference for the comparison and interpretation of the results. If the mitogen-stimulated value passes the cutoff value, it can confirm the host’s immune status for the IGRA interpretation. In humans, low mitogen responses found in IGRA test were most often associated with reduced lymphocyte activities due to insufficient lymphocytes, incorrect addition of the mitogen, prolonged specimen transport, improper specimen handling, or the presence of antibodies to IFN-γ [32–35]. In NHPs, two commercial TB-IGRA kits, PrimaGAM and gamma-interferon test (GIFT) assay, used different mitogens to stimulate lymphocytes. In PrimaGAM, Con A was used as a positive control mitogen [36], while a combination of Con A+PWM mitogens was used as a positive control in the GIFT assay [37]. However, the concentration and minimal response to Con A were not determined. Apart from Con A and PWM, PHA was also used as a mitogen for IGRA, with reports of inappropriate stimulation of immune cells in cynomolgus monkeys and chacma baboons [33, 38]. To date, no suitable mitogen for the IGRA test in cynomolgus monkeys has been widely recommended. Since MTBC infection can lead to immunosuppression, it is essential to use appropriate mitogens to ensure that the animal is immune competent enough to rule out false negative responding in the IGRA test. Here, we tested four different sets of mitogens, compared with a commercially available QFT-PHA mitogen and applied in the mIGRA assay for interpretation. Moreover, a new mIGRA interpretation algorithm was also proposed, based on IFN-γ levels as OD_450_ and selected mitogens, for TB diagnosis in cynomolgus macaques.

Although mitogen stimulation results depended heavily on the concentration used and the varied doses (dose-response) of mitogen were suggested in the test [20, 39], the high blood volume needed from each animal was a limitation. In this study, we selected four mitogens at a fixed dose of 20 μg/ml for the stimulation of IFN-γ release from T cells of cynomolgus monkeys, and the results were ranked as follows: Con A+PWM > PWM > QFT-PHA > Con A > PHA. Our results aligned with the previous study indicating that the QFT-PHA mitogen could activate higher IFN-γ secretion from the whole blood of cynomolgus macaques compared to Con A [20]. It should be noted that the concentration of Con A used in the previous study (5 μg/ml) was lower than that used in our study (20 μg/ml). Compared to the previous study in human PBMC isolated from healthy donors, a same order of stimulation was observed, although the concentrations of mitogens were lower than those used in our study: PWM (5 μg/ml) > PHA (10 μg/ml) > Con A (5μg/ml) [25]. A slight difference was observed in other studies using human whole blood derived from healthy volunteers, and a preferential order was PWM (2 μg/ml) ≥ Con A (5 μg/ml) ≥ QFT-GIT-PHA (the PHA mitogen from the QuantiFERON Gold kit) > PHA-P (10 μg/ml) [20, 40]. This suggests that mitogen stimulation results depend on the concentration used, cell density, and culture period [39]. When a constant number of lymphocytes was used, the dose-response sigmoid curve was usually observed. Stimulation tends to decrease at very high mitogen concentrations, which exceeds the optimal level, presumably due to nonspecific toxicity to the cells. Focusing on two types of PHA mitogens used in our study, the QFT-PHA mitogen stimulated IFN-γ secretion from the whole blood of cynomolgus macaques more effectively than the prepared PHA (20 μg/ml). This suggested that the concentration of PHA used in the QFT kit might exceed 20 μg/ml. Similar to the report in human whole blood, the commercial QFT-GIT-PHA mitogen stimulated human immune cells to secrete higher IFN-γ levels than that of the prepared PHA-P mitogen (20 μg/ml) [20]. The prepared PHA in our study was also of the PHA-P form, while the forms used in the QFT-GIT or QFT-plus kit remain unspecified. Commercially, two forms of PHA are available, the PHA-P protein form and the PHA-M mucoprotein form. We suspect that the higher IFN-γ response observed after QFT-PHA stimulation, compared to prepared PHA stimulation, could be attributed to either a higher dose of mitogen or a different form of PHA used.

The different actions of mitogens in stimulating human immune cells were attributed to variation in specific noncatalytic carbohydrate recognition domains (or glycoproteins) that influence their biochemical and physicochemical properties, carbohydrate binding specificity, and biological activities. The specific glycol-conjugated domain of mitogens bound to its primary target in the carbohydrate moiety of cell membrane glycoproteins known as the Toll-like receptors (TLRs). Con A bound to α-D-mannosyl residues, while PWM and PHA bound to N-acetyl-D-glucosamine [41]. In humans, PHA stimulated TLRs (-2/6, -4, and -5) on CD^3+^/CD^8+^ T cells. Con A bound to TLRs (-2/6) on CD^4+^/CD^8+^ T cells and exhibited higher mitogenic activity in CD^4+^ T cells compared to CD^8+^ T cells, resulting in enhanced proliferation and IFN-γ production [25]. PWM not only stimulated the TLRs (-2/6, -4, and -5) in CD3 + / CD4 + / CD8 + T cells, but also activated signals from TLR4 proteins in the B cells, leading to a proliferative response and IFN-γ production in both T and B cells [42]. This might explain why PWM had stronger mitogenic activity and stability compared to PHA and Con A in our study. The combination of Con A and PWM might enhance the number of specific TLRs on the surface of immune cells, resulting in higher levels of IFN-γ following a mixture of ConA and PWM stimulation compared to the use of a single mitogen. No indeterminate results were encountered when a mixture of Con A and PWM was used as the positive mitogen control for mIGRA interpretation, in contrast to the single mitogen usage, which results in two indeterminate cases for Con A and an indeterminate case for PWM.

In addition to a mitogen type, heat or cation-supplemented mitogens (Mg^2+^, Ca^2+^ and Zn^2+^) could affect IFN-γ production [20]. If the animals were supplemented with cations such as calcium, the detection of tuberculosis using the IGRA test should be considered. As shown in Fig 1, IFN-γ production in response to each mitogen varied among individual monkeys; some exhibited high responses, depending on their host immunity status. Therefore, this variability should be taken as awareness during diagnosis.

It is important to note that responses to mitogen stimulations might differ among various animal species. For example, PWM and PHA showed the same activity in inducing IFN-γ secretion from the whole blood cells of wild dogs [43]. Different positive control mitogens were suggested, such as PWM for domestic cattle (*Bos taurus*) [44], Con A for Asian elephants (*Elephas maximus*) [45], and PHA for human (QFT-plus, QIAGEN). Additionally, genetic differences among individuals within the same cohort should also be considered. When comparing IFN-γ secretion between QFT-PHA and Con A+PWM stimulations, 93% of cynomolgus macaques showed higher levels of IFN-γ after ConA + PWM stimulation than after QFT-PHA, while it was opposite for 5% of the animals. We hypothesized that the host’s genetic profile might lead to variations in specific membrane glycoproteins on immune cells. Therefore, factors such as animal species, genetic background, sample type (whole blood or PBMC), and the IGRA protocol (such as the duration of blood collection before testing, blood handling, and stimulation period) should be considered while performing the IGRA test.

When the positive mitogen control was changed from QFT-PHA to Con A+PWM along with the implementation of a new mIGRA interpretation algorithm to determine TB infection in a large cohort (100 monkeys, 8 rounds), the number of negative and indeterminate cases were remarkably noticed. Focusing on the number of indeterminate cases in the condition of OD_MIT-NIL_ ≤ 0.18, it was significantly reduced (80–100%). Unfortunately, although the mitogen was changed, the number of indeterminate, causing from OD_NIL_>0.18, remained unchanged. This implied that the latter group of indeterminate animals may have underlying health issues or inflammation, leading to have high γ-IFN backgrounds, which should be considered for follow-up. Therefore, this suggests that the Con A+PWM mitogen should be applied to mIGRA for the detection of tuberculosis in cynomolgus monkeys. The reduction in indeterminate cases would also decrease the number of animals undergoing euthanasia or being in the quarantine programs. However, the indeterminate cases remained in the Con A+PWM group. Possible explanations could be (1) an insufficient immune response to the mitogens due to the impaired host immune system, such as suspected immunosuppression, chronic disease, young age, and malnutrition; (2) technical errors (e.g., delays in sample delivery or vigorous handling) during sample collection; or (3) anergy. When the indeterminate cases were detected by mIGRA, it was likely that the host was MTBC-infected, and those cynomolgus macaques should be monitored, with additional assays used as ancillary tests. Several TB detection methods, based on the cellular immune response as seen in our mIGRA, were GIFT, T-Spot, or ELISpot, and TST. They differed in the testing formats (*in vitro* whole blood tests for mIGRA and GIFT; *in vitro* PMBC test for T-Spot and ELISpot; *in vivo* test for TST), the antigens used (specific TB peptide antigens for mIGRA; bovine-PPD and avian- PPD for GIFT; and MOT-PPD for TST), and the interpretation criteria [9, 13, 16, 37]. Thus, the test results might not be interchangeable [34]. In conclusion, a single diagnostic tool was not recommended for TB screening in cynomolgus macaques; rather, a combination of TB diagnostic tests such as PCR, IGRA, TST, and antibody-ELISA should be considered [11].

## Supporting information

S1 Table The IFN-γ values of individual 12 selected cynomolgus macaques after the subtraction of plasma and IFN-γ (NIL) background, as OD_MIT-NIL_. All values were obtained after stimulation with prepared PHA (20 μg/ml), Con A (20 μg/ml), PWM (20 μg/ml), Con A+PWM (20 μg/ml each), and (QFT-PHA). Red highlighted indicated the values below the 95^th^ percentile of the OD_NIL_ (= 0.18).
(DOCX)

S2 Table mIGRA interpretation of 100 cynomolgus macaques at month-0 (M0), month-4 (M4), month-7 (M7), month-14 (M14), month-17 (M17), month-20 (M20), month-26 (M26), and month-29 (M29), sorting by groups as active-TB, latent-TB and healthy based on results of GeneXpert MTB/RIF Ultra assay [29], mIGRA, TST(data not shown) and antibody test (data not shown). The interpretation was done by using an algorithm as described in Fig 3. A mixture of ConA and PWM, and QFT-PHA were used as a mitogen for interpretation as indicated. *indicates a time-point when the positive result of the GeneXpert MTB/RIF Ultra assay was reported [29]. N/A means not applicable. Pos, Neg and ID mean positive, negative, and indeterminate to mIGRA test, respectively.
(DOCX)
