# Supplementary material for: The new gamma interferon (IFN-γ) algorithm for tuberculosis diagnosis in cynomolgus macaques
Source: PLoS One. 2024 Dec 16;19(12):e0302349. doi: 10.1371/journal.pone.0302349 (PMC11649081; doi:10.1371/journal.pone.0302349)
Supplement: S1 Table — All values were obtained after stimulation with prepared PHA (20 μg/ml), Con A (20 μg/ml), PWM (20 μg/ml), Con A+PWM (20 μg/ml each), and (QFT-PHA). Red highlighted indicated the values below the 95th percentile of the ODNIL (= 0.18). (DOCX) [file pone.0302349.s001.docx]

**Supporting Information**

**S1 Table.** The IFN-γ values of 12 selected cynomolgus macaques after the subtraction of plasma and IFN-γ (NIL) background**,** as OD_MIT-NIL_. All values were obtained after stimulation with prepared PHA (20 μg/ml), Con A (20 μg/ml), PWM (20 μg/ml), Con A+PWM (20 μg/ml each), and (QFT-PHA). Red highlighted indicated the values below the 95^th^ percentile of the OD_NIL_ (= 0.18).

| **Monkey KBK#** | **Absorbance values at 450nm (OD_MIT-NIL_)** | | | | |
| --- | --- | --- | --- | --- | --- |
|  | **PHA** | **Con A** | **PWM** | **Con A +PWM** | **(QFT-PHA)** |
| **035** | 0.084 | 0.312 | 3.780 | 4.316 | 1.980 |
| **066** | 0.490 | 0.746 | 3.237 | 3.604 | 1.785 |
| **069** | 0.289 | 0.205 | 3.127 | 3.984 | 0.322 |
| **097** | 0.035 | 0.285 | 3.382 | 3.763 | 0.350 |
| **114** | -0.027 | -0.021 | 0.101 | 0.258 | 0.076 |
| **116** | 0.073 | 0.364 | 3.610 | 3.798 | 0.998 |
| **117** | 0.017 | 0.034 | 2.843 | 3.619 | 0.359 |
| **118** | 0.157 | 0.338 | 3.766 | 4.349 | 2.696 |
| **119** | 0.593 | 0.479 | 3.669 | 4.100 | 0.790 |
| **193** | 2.051 | 2.227 | 3.587 | 3.662 | 2.197 |
| **292** | 0.414 | 1.081 | 3.584 | 4.270 | 3.860 |
| **300** | 0.149 | 2.849 | 3.733 | 3.827 | 2.300 |
