# Supplementary material for: The new gamma interferon (IFN-γ) algorithm for tuberculosis diagnosis in cynomolgus macaques
Source: PLoS One. 2024 Dec 16;19(12):e0302349. doi: 10.1371/journal.pone.0302349 (PMC11649081; doi:10.1371/journal.pone.0302349)
Supplement: S2 Table — The interpretation was done by using an algorithm as described in Fig 3. A mixture of ConA and PWM, and QFT-PHA were used as a mitogen for interpretation as indicated. *indicates a time-point when the positive result of the GeneXpert MTB/RIF Ultra assay was reported [29]. N/A means not applicable. Pos, Neg and ID mean positive, negative, and indeterminate to mIGRA test, respectively. (DOCX) [file pone.0302349.s002.docx]

**Supporting Information**

**S2 Table:** mIGRA interpretation of 100 cynomolgus macaques at month-0 (M0), month-4 (M4), month-7 (M7), month-14 (M14), month-17 (M17), month-20 (M20), month-26 (M26), and month-29 (M29), sorting by groups as active-TB, latent-TB and healthy based on results of GeneXpert MTB/RIF Ultra assay [29], mIGRA, TST(data not shown) and antibody-ELISA test (data not shown). The interpretation was done by using an algorithm as described in **Fig 3**. A mixture of ConA and PWM, and QFT-PHA were used as a mitogen for interpretation as indicated. *indicates a time-point when the positive result of the GeneXpert MTB/RIF Ultra assay was reported [29]. N/A means not applicable. Pos, Neg and ID mean positive, negative, and indeterminate to mIGRA test, respectively.

| **Group** | **Monkey KBK no.** | **mIGRA interpretation when a mixture of ConA and PWM was used as a mitogen** | | | | | | | |  | **Monkey KBK no.** | **mIGRA interpretation when QFT-PHA was used as a mitogen** | | | | | | | | |
| --- | --- | --- | --- | --- | --- | --- | --- | --- | --- | --- | --- | --- | --- | --- | --- | --- | --- | --- | --- | --- |
|  |  | **M0** | **M4** | **M7** | **M14** | **M17** | **M20** | **M26** | **M29** |  |  | **M0** | **M4** | **M7** | **M14** | **M17** | **M20** | **M26** | **M29** |  |
| Active-TB | **99** | Neg | Pos * | ID***** | death of TB; lung and liver culture in M9 | death | death | death | death |  | **99** | ID | Pos * | ID***** | death of TB; lung and liver culture in M9 | death | death | death | death |  |
|  | **103** | Pos * | Pos * | ID | death of TB; lung and liver culture in M10 | death | death | death | death |  | **103** | Pos * | Pos * | ID | death of TB; lung and liver culture in M10 | death | death | death | death |  |
|  | **293** | ID | ID***** | ID***** | death of TB; lung and liver culture in M10 | death | death | death | death |  | **293** | ID | ID***** | ID***** | death of TB; lung and liver culture in M10 | death | death | death | death |  |
|  | **102** | Neg* | Neg | Neg | Neg | Neg | N/A | death | death |  | **102** | Neg* | Neg | Neg | ID | ID | N/A | death | death |  |
|  | **46** | Neg* | Neg | death | death | death | death | death | death |  | **46** | Neg* | Neg | death | death | death | death | death | death |  |
|  | **49** | Neg | Neg* | Neg | Neg | Neg | Neg | Neg | Neg |  | **49** | Neg | Neg* | Neg | Neg | Neg | Neg | Neg | ID |  |
|  | **95** | Pos | ID | Pos | Pos | ID | ID | ID***** | ID |  | **95** | Pos | ID | Pos | Pos | ID | ID | ID***** | ID |  |
|  | **96** | Neg* | N/A | Pos | Pos | Pos | Pos | Neg | Pos |  | **96** | Neg* | N/A | Pos | Pos | Pos | Pos | Neg | Pos |  |
|  | **100** | Pos | Pos | Neg | Neg* | Neg | Neg | Neg | Neg |  | **100** | Pos | Pos | Neg | Neg* | Neg | ID | Neg | Neg |  |
|  | **104** | Neg* | Neg | Neg | Neg | Neg | Neg | Neg | Neg |  | **104** | ID***** | Neg | Neg | Neg | Neg | Neg | Neg | Neg |  |
|  | **105** | Pos * | Pos | Pos | Pos | Pos | Pos | ID | ID |  | **105** | Pos * | Pos | Pos | Pos | Pos | Pos | ID | ID |  |
|  | **120** | Neg* | Neg | Neg | Neg | Neg | Pos | Neg | Pos |  | **120** | ID***** | ID | ID | ID | ID | Pos | ID | Pos |  |
|  | **195** | Pos* | Pos | Pos | ID | ID | ID | ID | Pos |  | **195** | Pos* | Pos | Pos | ID | ID | ID | ID | Pos |  |
|  | **199** | Neg* | Neg | Neg | Pos | Neg | Neg | Neg | Neg |  | **199** | ID***** | Neg | ID | Pos | Neg | Neg | ID | ID |  |
|  | **264** | Neg* | Neg | Neg | Neg | Neg | Neg | Neg | Neg |  | **264** | Neg* | Neg | Neg | Neg | Neg | Neg | Neg | Neg |  |
|  | **266** | Neg* | Pos | Neg | Neg | Neg | Neg | Neg | Neg |  | **266** | ID***** | Pos | ID | Neg | Neg | Neg | ID | ID |  |
|  | **292** | Neg | Neg | Neg* | Neg | Pos | Neg | Neg | Neg |  | **292** | Neg | Neg | Neg* | Neg | Pos | Neg | Neg | Neg |  |
|  | **294** | Pos | Pos | Pos | Pos | Pos | ID | ID***** | ID |  | **294** | Pos | Pos | Pos | Pos | Pos | ID | ID***** | ID |  |
|  | **298** | Neg* | Neg | ID | Neg | Neg | Neg | Neg | Neg |  | **298** | Neg* | Neg | ID | Neg | Neg | Neg | Neg | Neg |  |
|  |  |  |  |  |  |  |  |  |  |  |  |  |  |  |  |  |  |  |  |  |
| Latent-TB | **35** | Pos | Neg | Neg | Neg | Neg | Neg | Neg | Neg |  | **35** | Pos | Neg | Neg | Neg | Neg | Neg | Neg | Neg |  |
|  | **36** | Pos | Neg | Neg | Neg | Neg | Neg | Neg | Neg |  | **36** | Pos | ID | Neg | ID | ID | Neg | ID | ID |  |
|  | **38** | Pos | Neg | Neg | Neg | Neg | Neg | Neg | Neg |  | **38** | Pos | Neg | Neg | Neg | Neg | Neg | Neg | Neg |  |
|  | **40** | Pos | Neg | Neg | Neg | Neg | Neg | Neg | Neg |  | **40** | Pos | Neg | Neg | Neg | Neg | Neg | Neg | Neg |  |
|  | **42** | Pos | Neg | Neg | Neg | Neg | Neg | Neg | Neg |  | **42** | Pos | Neg | Neg | Neg | Neg | Neg | ID | ID |  |
|  | **45** | Neg | Pos | Pos | N/A | Pos | Pos | Pos | Neg |  | **45** | Neg | Pos | Pos | N/A | Pos | Pos | Pos | Neg |  |
|  | **48** | Pos | Pos | Pos | Pos | Pos | Pos | Pos | Pos |  | **48** | Pos | Pos | Pos | Pos | Pos | Pos | Pos | Pos |  |
|  | **54** | Neg | Neg | Neg | Neg | Pos | Neg | Neg | Neg |  | **54** | Neg | Neg | Neg | Neg | Pos | Neg | Neg | Neg |  |
|  | **55** | Pos | N/A | Pos | Pos | Pos | Pos | Neg | Neg |  | **55** | Pos | N/A | Pos | Pos | Pos | Pos | Neg | Neg |  |
|  | **56** | Neg | Pos | Neg | Neg | Pos | N/A | Neg | Neg |  | **56** | ID | Pos | Neg | Neg | Pos | N/A | ID | Neg |  |
|  | **57** | Neg | Pos | Neg | death | death | death | death | death |  | **57** | Neg | Pos | Neg | death | death | death | death | death |  |
|  | **60** | Neg | Neg | Neg | Neg | Neg | Pos | Neg | Neg |  | **60** | Neg | Neg | Neg | Neg | Neg | Pos | Neg | Neg |  |
|  | **65** | ID | Neg | Neg | death | death | death | death | death |  | **65** | ID | Neg | Neg | death | death | death | death | death |  |
|  | **87** | Neg | Neg | Neg | Neg | Neg | Neg | Neg | Neg |  | **87** | Neg | Neg | Neg | Neg | Neg | Neg | Neg | Neg |  |
|  | **89** | Neg | Neg | Neg | Pos | Pos | Pos | Neg | Neg |  | **89** | Neg | Neg | Neg | Pos | Pos | Pos | Neg | Neg |  |
|  | **90** | Neg | Neg | Neg | Neg | Neg | Neg | Neg | Neg |  | **90** | ID | Neg | Neg | ID | ID | Neg | ID | Neg |  |
|  | **92** | Neg | Pos | Pos | Pos | Pos | ID | Pos | Neg |  | **92** | Neg | Pos | Pos | Pos | Pos | ID | Pos | Neg |  |
|  | **93** | Pos | Pos | Pos | Pos | Pos | Pos | Pos | Pos |  | **93** | Pos | Pos | Pos | Pos | Pos | Pos | Pos | Pos |  |
|  | **98** | Neg | Pos | Neg | Neg | Neg | Neg | Neg | ID |  | **98** | Neg | Pos | Neg | Neg | Neg | Neg | Neg | ID |  |
|  | **101** | Neg | Pos | death | death | death | death | death | death |  | **101** | ID | Pos | death | death | death | death | death | death |  |
|  | **169** | Neg | Neg | Neg | Neg | death | death | death | death |  | **169** | Neg | Neg | Neg | Neg | death | death | death | death |  |
|  | **170** | Neg | Neg | Neg | death | death | death | death | death |  | **170** | Neg | Neg | Neg | death | death | death | death | death |  |
|  | **171** | Neg | Neg | Neg | Neg | Neg | Neg | Neg | Neg |  | **171** | Neg | Neg | Neg | Neg | Neg | Neg | Neg | Neg |  |
|  | **173** | Pos | Neg | Neg | Neg | Neg | Neg | Pos | Neg |  | **173** | Pos | ID | ID | ID | ID | Neg | Pos | ID |  |
|  | **175** | Neg | Neg | Neg | Neg | Neg | Neg | Neg | Neg |  | **175** | Neg | Neg | Neg | Neg | Neg | Neg | Neg | Neg |  |
|  | **193** | Neg | Neg | Neg | Neg | Neg | Pos | Pos | Neg |  | **193** | Neg | Neg | Neg | Neg | Neg | Pos | Pos | Neg |  |
|  | **194** | Neg | Neg | Neg | Neg | Neg | Neg | Neg | Neg |  | **194** | Neg | Neg | Neg | Neg | Neg | Neg | Neg | Neg |  |
|  | **196** | Neg | Neg | Neg | Pos | Neg | Neg | Neg | Neg |  | **196** | Neg | Neg | Neg | Pos | Neg | Neg | Neg | Neg |  |
|  | **198** | Neg | Neg | Neg | Neg | Neg | Neg | Pos | Neg |  | **198** | Neg | Neg | Neg | Neg | Neg | Neg | Pos | Neg |  |
|  | **200** | Pos | Neg | Neg | death | death | death | death | death |  | **200** | Pos | Neg | Neg | death | death | death | death | death |  |
|  | **262** | Neg | Neg | Neg | Pos | Pos | Pos | Neg | Pos |  | **262** | Neg | Neg | Neg | Pos | Pos | Pos | Neg | Pos |  |
|  | **265** | Neg | Pos | Pos | Pos | Pos | Pos | Neg | Neg |  | **265** | ID | Pos | Pos | Pos | Pos | Pos | ID | ID |  |
|  | **268** | Pos | ID | Pos | Pos | Pos | Pos | Pos | Pos |  | **268** | Pos | ID | Pos | Pos | Pos | Pos | Pos | Pos |  |
|  | **300** | Neg | Neg | Pos | Neg | Neg | Neg | Neg | Neg |  | **300** | Neg | Neg | Pos | Neg | Neg | Neg | ID | Neg |  |
|  |  |  |  |  |  |  |  |  |  |  |  |  |  |  |  |  |  |  |  |  |
|  | **51** | Neg | Neg | death | death | death | death | death | death |  | **51** | Neg | Neg | death | death | death | death | death | death |  |
|  | **52** | Neg | Neg | Neg | death | death | death | death | death |  | **52** | ID | Neg | Neg | death | death | death | death | death |  |
|  | **114** | Neg | Neg | Neg | Neg | Neg | death | death | death |  | **114** | ID | Neg | Neg | Neg | ID | death | death | death |  |
| Healthy | **168** | Neg | Neg | death | death | death | death | death | death |  | **168** | Neg | Neg | death | death | death | death | death | death |  |
|  | **86** | Neg | Neg | Neg | loss | loss | loss | loss | loss |  | **86** | Neg | Neg | Neg | loss | loss | loss | loss | loss |  |
|  | **31** | Neg | Neg | Neg | Neg | Neg | Neg | Neg | Neg |  | **31** | ID | Neg | Neg | Neg | Neg | Neg | ID | ID |  |
|  | **32** | Neg | Neg | Neg | Neg | Neg | Neg | Neg | Neg |  | **32** | ID | Neg | Neg | Neg | Neg | Neg | ID | Neg |  |
|  | **33** | Neg | Neg | Neg | Neg | Neg | Neg | Neg | Neg |  | **33** | Neg | ID | Neg | Neg | ID | Neg | ID | Neg |  |
|  | **34** | Neg | Neg | Neg | Neg | Neg | Neg | Neg | Neg |  | **34** | Neg | Neg | Neg | Neg | Neg | Neg | Neg | Neg |  |
|  | **37** | Neg | Neg | Neg | Neg | Neg | Neg | Neg | Neg |  | **37** | Neg | Neg | Neg | Neg | Neg | Neg | Neg | Neg |  |
|  | **39** | Neg | ID | Neg | Neg | Neg | Neg | Neg | Neg |  | **39** | ID | ID | Neg | Neg | Neg | Neg | Neg | Neg |  |
|  | **41** | Neg | Neg | Neg | Neg | Neg | Neg | Neg | Neg |  | **41** | ID | Neg | Neg | Neg | ID | Neg | ID | Neg |  |
|  | **43** | ID | Neg | Neg | Neg | Neg | Neg | Neg | Neg |  | **43** | ID | Neg | ID | ID | ID | ID | ID | ID |  |
|  | **44** | ID | Neg | Neg | Neg | Neg | Neg | Neg | Neg |  | **44** | ID | Neg | Neg | Neg | Neg | Neg | Neg | Neg |  |
|  | **47** | Neg | Neg | Neg | Neg | Neg | Neg | Neg | Neg |  | **47** | Neg | Neg | Neg | ID | Neg | Neg | Neg | Neg |  |
|  | **50** | Neg | Neg | Neg | Neg | Neg | Neg | Neg | Neg |  | **50** | ID | Neg | Neg | ID | Neg | Neg | ID | Neg |  |
|  | **53** | Neg | Neg | Neg | Neg | Neg | Neg | Neg | Neg |  | **53** | ID | Neg | Neg | ID | Neg | Neg | ID | Neg |  |
|  | **58** | Neg | Neg | Neg | Neg | Neg | Neg | Neg | Neg |  | **58** | ID | Neg | Neg | ID | ID | Neg | ID | Neg |  |
|  | **59** | Neg | Neg | Neg | Neg | Neg | Neg | Neg | Neg |  | **59** | ID | Neg | Neg | Neg | ID | Neg | ID | Neg |  |
|  | **61** | Neg | Neg | Neg | Neg | Neg | Neg | Neg | Neg |  | **61** | ID | Neg | Neg | ID | ID | Neg | Neg | ID |  |
|  | **62** | Neg | Neg | Neg | Neg | Neg | Neg | Neg | Neg |  | **62** | Neg | Neg | Neg | Neg | Neg | Neg | Neg | Neg |  |
|  | **63** | Neg | Neg | Neg | Neg | Neg | N/A | Neg | Neg |  | **63** | ID | Neg | Neg | Neg | Neg | N/A | Neg | Neg |  |
|  | **64** | ID | Neg | Neg | Neg | Neg | Neg | Neg | Neg |  | **64** | ID | Neg | ID | ID | ID | ID | ID | ID |  |
|  | **66** | Neg | Neg | Neg | Neg | Neg | Neg | Neg | Neg |  | **66** | Neg | Neg | Neg | Neg | Neg | Neg | Neg | Neg |  |
|  | **67** | Neg | Neg | Neg | Neg | Neg | Neg | Neg | Neg |  | **67** | Neg | Neg | Neg | Neg | Neg | Neg | Neg | Neg |  |
|  | **68** | Neg | Neg | Neg | Neg | Neg | Neg | Neg | Neg |  | **68** | Neg | Neg | Neg | Neg | Neg | Neg | Neg | Neg |  |
| Healthy | **69** | ID | Neg | Neg | Neg | Neg | Neg | Neg | Neg |  | **69** | ID | Neg | ID | Neg | Neg | Neg | ID | ID |  |
|  | **70** | Neg | Neg | Neg | Neg | Neg | Neg | Neg | Neg |  | **70** | Neg | ID | Neg | Neg | Neg | Neg | Neg | Neg |  |
|  | **72** | Neg | Neg | Neg | Neg | Neg | Neg | Neg | Neg |  | **72** | Neg | Neg | Neg | Neg | Neg | Neg | Neg | Neg |  |
|  | **83** | Neg | Neg | Neg | Neg | Neg | Neg | Neg | Neg |  | **83** | ID | Neg | Neg | ID | ID | Neg | ID | Neg |  |
|  | **84** | Neg | Neg | Neg | Neg | Neg | Neg | Neg | Neg |  | **84** | Neg | Neg | Neg | Neg | Neg | Neg | Neg | Neg |  |
|  | **85** | Neg | Neg | Neg | Neg | Neg | Neg | Neg | Neg |  | **85** | Neg | Neg | Neg | Neg | Neg | Neg | Neg | Neg |  |
|  | **88** | Neg | Neg | Neg | Neg | Neg | Neg | Neg | Neg |  | **88** | ID | Neg | Neg | Neg | ID | Neg | Neg | Neg |  |
|  | **94** | ID | Neg | Neg | Neg | Neg | Neg | Neg | Neg |  | **94** | ID | ID | Neg | Neg | ID | ID | ID | Neg |  |
|  | **97** | Neg | Neg | Neg | Neg | Neg | Neg | Neg | Neg |  | **97** | ID | Neg | Neg | Neg | Neg | Neg | Neg | Neg |  |
|  | **115** | Neg | Neg | Neg | Neg | Neg | Neg | Neg | Neg |  | **115** | Neg | Neg | Neg | Neg | Neg | Neg | ID | ID |  |
|  | **116** | Neg | Neg | Neg | Neg | Neg | Neg | Neg | Neg |  | **116** | Neg | Neg | Neg | Neg | Neg | Neg | Neg | Neg |  |
|  | **117** | Neg | Neg | Neg | Neg | Neg | Neg | Neg | Neg |  | **117** | ID | ID | ID | ID | Neg | ID | ID | ID |  |
|  | **118** | Neg | Neg | Neg | Neg | Neg | Neg | Neg | Neg |  | **118** | Neg | Neg | Neg | Neg | Neg | Neg | ID | Neg |  |
|  | **119** | Neg | Neg | Neg | Neg | Neg | Neg | Neg | Neg |  | **119** | Neg | Neg | Neg | Neg | Neg | Neg | Neg | ID |  |
|  | **172** | Neg | Neg | Neg | Neg | Neg | Neg | Neg | Neg |  | **172** | Neg | Neg | Neg | ID | Neg | Neg | Neg | Neg |  |
|  | **174** | Neg | Neg | Neg | Neg | Neg | Neg | Neg | Neg |  | **174** | ID | Neg | ID | ID | Neg | Neg | ID | ID |  |
|  | **197** | Neg | Neg | Neg | Neg | Neg | Neg | Neg | Neg |  | **197** | Neg | Neg | Neg | Neg | Neg | Neg | Neg | Neg |  |
|  | **263** | Neg | Neg | Neg | Neg | Neg | Neg | Neg | Neg |  | **263** | Neg | Neg | ID | Neg | Neg | Neg | ID | ID |  |
|  | **267** | Neg | Neg | Neg | Neg | Neg | Neg | Neg | Neg |  | **267** | Neg | Neg | Neg | Neg | Neg | Neg | Neg | Neg |  |
|  | **269** | Neg | Neg | Neg | Neg | Neg | Neg | Neg | Neg |  | **269** | ID | ID | ID | Neg | ID | ID | ID | ID |  |
|  | **299** | Neg | Neg | Neg | Neg | Neg | Neg | Neg | Neg |  | **299** | Neg | Neg | Neg | Neg | Neg | Neg | Neg | Neg |  |
